# Is IDDSI an Evidence-Based Framework? A Relevant Question for the Frail Older Population

**DOI:** 10.3390/geriatrics5040082

**Published:** 2020-10-21

**Authors:** Claudia Côté, Amélie Giroux, Annie Villeneuve-Rhéaume, Cynthia Gagnon, Isabelle Germain

**Affiliations:** 1 Centre de Recherche Charles-Le Moyne-Saguenay-Lac-Saint-Jean sur les Innovations en Santé (CR-CSIS), Faculty of Medicine and Health Sciences, University of Sherbrooke, Sherbrooke, QC J1H 5N4, Canada; claudia.cote4@usherbrooke.ca (C.C.); annie.villeneuve-rheaume@usherbrooke.ca (A.V.-R.); cynthia.gagnon4@usherbrooke.ca (C.G.); 2 Groupe de Recherche Interdisciplinaire sur les Maladies Neuromusculaires (GRIMN), Centre Intégré Universitaire de Santé et de Services Sociaux du Saguenay-Lac-Saint-Jean, Jonquière, QC G7X 7X2, Canada; 3 Association Professionnelle des Nutritionnistes Experts en Dysphagie (APNED), CP 76555, CP Buies, Montréal, QC H1S 0C9, Canada; information@apned.info; 4 Saint-Hyacinthe Research and Development Centre, Agriculture and Agri-Food Canada, 3600 Boul. Casavant West, Saint-Hyacinthe, QC J2S 8E3, Canada; 5 School of Human Nutrition, McGill University, 21, 111 Lakeshore Road, Sainte-Anne-de-Bellevue, QC H9X 3L9, Canada

**Keywords:** IDDSI, dysphagia, swallowing, thickened liquids, texture modified food, nutrition, older adults, metrological qualities, COSMIN

## Abstract

To delay impacts of aging, optimal nutritional status is essential. Several factors can reduce food intake, such as isolation, income, and cognitive/physical decline. Additionally, chewing and swallowing difficulties, or dysphagia, often disrupt the ability to consume life-long favorite dishes. Food and liquids could require modification of texture or consistency to ensure a comfortable or safe swallow. The food industry, foodservices facilities, and caregivers need quality control benchmarks to provide adequate nourishment and meet these new feeding challenges. The International Dysphagia Diet Standardisation Initiative (IDDSI) is proposing the IDDSI framework and testing methods to describe food used in nutritional care plans to circumvent dysphagia and improve communication among caregivers. This systematic review assesses the validity and reliability of the IDDSI testing methods using the Consensus-based Standards for the selection of health Measurement Instruments (COSMIN). Two publications presented content validity whereas 19 publications looked at construct validity or reliability for the IDDSI testing methods. One study was conducted in older adults presenting dysphagia. This review concludes that there is insufficient evidence to recommend the IDDSI testing methods. Further research, conducted with robust methodological design and reporting, is needed to develop and assess nutritious adapted food for frail older populations.

## 1. Introduction

**Figure 1:**
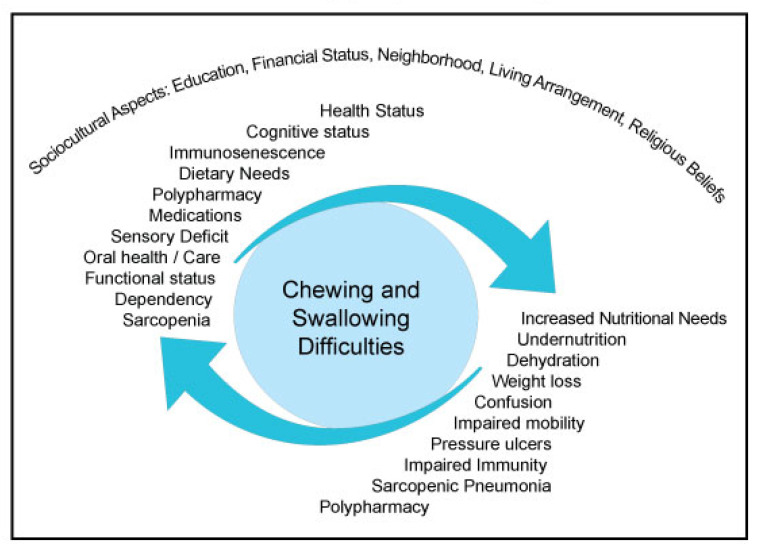
Interaction model of key factors affecting feeding in the context of dysphagia in the older adult population.

To avoid or delay health decline associated to aging, appropriate nutritional intake is frequently an essential part of the solution. Whether in community dwellings or in long-term facilities, several factors can hinder food intake: psycho-social changes (loneliness, depression), financial stress, physiological changes in taste and smell, polypharmacy, impaired digestion/absorption of nutrients, mobility decline, as well as chronic diseases [1,2,3]. Furthermore, food intake could be affected by poor oral health, difficulty in chewing or swallowing—dysphagia—which has been associated to undernutrition [4,5,6]. In the complex geriatric context, Okazaki and colleagues (2019) recently presented the concept of “vicious circle of aspiration pneumonia” where sarcopenia, undernutrition, and inflammation are at the core of recurrent aspiration pneumonia [7]. Sarcopenia is recognized as a syndrome characterized by a progressive and generalized loss of skeletal muscle mass and strength, with a risk of adverse outcomes, such as physical disability, poor quality of life and death [8]. Sarcopenic dysphagia and presbyphagia, defined as age-related changes in the swallowing mechanism, are investigated with more interest recently [9]. Deterioration in immune system functions, known as immunosenescence, is also well documented in the older adult population and is acerbated by poor nutritional status [10,11]. Sarcopenia and immunosenescence are presented as predisposing factors to aspiration pneumonia in frail older adult populations [12,13,14,15,16] further complicating the evaluation of the causes of recurrent aspiration pneumonia in this population. Providing nutritious and safe texture modified foods are key elements in mitigating dysphagia impacts (Figure 1).

Dysphagia is known to result in detrimental health effects and has been associated with overlooked undernutrition [17,18,19]. The cornerstone of dysphagia treatment is often the modification of food texture and liquid consistency [20]. Older adults presenting with dysphagia will often need to receive a diet that is adapted to the oropharyngeal structures affected and the severity of the dysphagia. The adaptation of food texture might require the consumption of softer, minced, or even pureed foods. The consistency of the liquids might need to be thickened. As appetite and salivation are stimulated by the appearance and the smell of foods, these modifications, although simple at first glance, will further complicate meal preparation and appreciation [21]. The texture modified foods are also known to vary in nutrient content, can be less appealing, and lead to reduced food intake or poor compliance [22,23,24,25,26,27]. Enrichment or fortification of pureed foods is suggested to compensate for poor intake or poor nutritional density [23,28,29], and individualized nutrition care plans should be considered. Finally, when foods are carefully selected, positive impact on food intakes and weight are observed [22]. Free water protocols can support hydration to improve hydration in well-defined populations [30,31]. To date, research looking at efficacy of the nutritional treatment to circumvent dysphagia is insufficient [20,32,33]. Quantifiable descriptors of food texture and liquid consistency, analysis of nutritional density of proposed food solutions with an improved and stimulating appealing look are necessary to bridge these gaps in scientific research.

For decades now, two main challenges have been identified with the adaptation of texture and consistency [34,35]. First, the description of the clinically adequate texture modified foods is problematic as bedside assessments are often limited to a few foods. Furthermore, foodstuff used in clinical assessments, such as via videofluoroscopy, are not always representative of mealtime diet. Secondly, food texture parameters are mainly subjectively assessed in clinical setting. Therefore, providing the incorrect food texture or consistency could put the older adults at risk of undernutrition/dehydration, recurrent airway infections due to penetration or aspiration of food particles or oral bacterial content, aspiration pneumonia or even suffocation. Recognizing these risks, health care professionals around the world have developed a significant number of dietary approaches with various descriptors (labels or qualifiers) as well as quality control assessments to secure food intake [20].

With the aging of the worldwide population, dysphagia in the older adults has gained more attention from the international scientific community. Hence, since 2013, the International Dysphagia Diet Standardisation Initiative (IDDSI) has developed a framework with the intent to globally harmonize the terminology used to describe food and liquids used in nutritional care plans developed to help manage dysphagia [36,37]. IDDSI descriptors for food and drinks are accompanied by visual aids and implementation guides for clinicians, food service and food industry available in more than 25 languages (www.iddsi.org). Two interconnected and color coded pyramids represent the proposed IDDSI progression of food and drinks, on continuum from level 0 to level 7 (Table 1). Several testing methods support the IDDSI framework, which include the IDDSI Flow test, Fork Drip test, the Spoon Tilt test, the Fork/Spoon pressure test, the Chopstick test, and the Finger test [38]. Although sound descriptors for dysphagia diet have long been awaited by clinical and research multidisciplinary teams, the implementation of particular measuring instruments must be justified based on its metrological properties. This publication intends to document the supporting evidence for the validation of the proposed IDDSI descriptors and metrological properties of the IDDSI testing methods in the geriatric context.

## 2. Materials and Methods

A systematic review of the publications regarding the validity and reliability of the IDDSI methods was carried out. Articles published up until July 2020, in English and French, were identified on different databases using a combination of keywords (Table 2). Two authors (I.G. and C.C.) selected the articles from the titles and abstracts and a third author validated the selection (C.G.). Disagreements were resolved by consensus. Guided by the PRISMA statement, articles were included if they used the framework proposed by the IDDSI or associated methods. Articles presenting no evidence of validity or reliability of IDDSI methods were rejected.

The COSMIN taxonomy and the COSMIN checklist (Consensus-based Standards for the selection of health status Measurement Instruments) were used to define measurement properties and as a guide to evaluate the methodological quality of the studies [39,40,41]. As a reminder, the validity of a method refers to the ability to truly measure what it must measure. According to COSMIN, the reliability is “the degree to which the measurement is free from measurement error”. Reliability refers to the ability to produce similar results, if the same user repeats the same method on the same product (repeatability) and under several conditions (reproducibility) [41]. It is usually expressed by means of a coefficient (intra-class correlation coefficient (ICC), kappa, etc.), whose value is between 0 and 1.

## 3. Results

**Figure 2:**
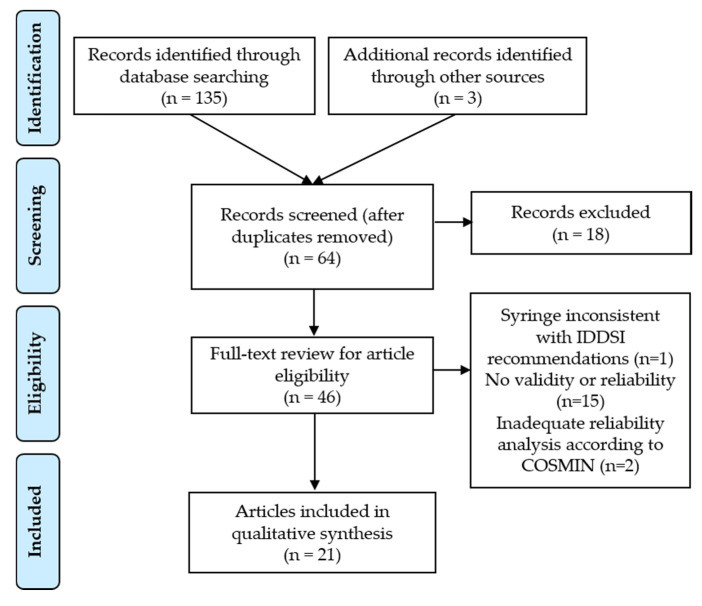
PRISMA Flow Diagram of systematic review process.

As a result of this systematic review, 21 publications were selected. No article was found to have assessed the metrological qualities of the IDDSI framework testing methods as their main objective. Nevertheless, publications presented elements of validity and/or reliability assessment. Figure 2 presents the result of the research strategy (PRISMA diagram). The selected studies are presented in Table 3. Research teams essentially experimented with the IDDSI flow test and primarily used water mixed with thickening agents. The type of thickening agent, preparation methods, temperature and resting time were variable. Most authors used liquids corresponding to a central value within each IDDSI level and not a set of values, which would have been more representative of the continuum. Precise flow values (amount of ml remaining after 10 s) are not always reported. Only one study recruited adult dysphagic participants [42]. According to our review, no studies have shown that the proposed terminology and levels improve the treatment of dysphagia, whether on acceptability, quality of life, nutritional status, hydration, or aspiration pneumonia. Finally, no publication has investigated the validity of IDDSI methods for measuring food texture.

### 3.1. Content Validity

The IDDSI framework was developed by an international and multidisciplinary group of experts [36,37]. The framework is based on scientific publications that suggest that changes in consistency or texture of food and liquids have a beneficial therapeutic effect related to the reduction of the risk of penetration/aspiration and choking [36,37]. The number of levels of food and beverage modification and the various testing methods were selected following a systematic review of the literature supplemented by an international consultation on existing dysphagia treatment practices. The selection of a syringe as a measuring tool was based on a theoretical rationale inspired by the Posthumus funnel, which presents technical specifications that are different to those of syringes [61,62]. No studies had assessed the syringe for its validity at the time of the publication of the IDDSI framework [37]. The proposed testing methods for evaluating the texture of foods and their theoretical rationales are discussed in an article, but the metrological qualities of these methods for the treatment of dysphagia are not documented [63].

### 3.2. Construct Validity

#### 3.2.1. Measurement Comparison of the IDDSI Flow Test with Other Related Tools

The IDDSI flow test was compared to other empirical methods (line-spread test and Bostwick consistometer) as well as rheological methods (viscometer and rheometer) to categorize various liquids. These methods showed that characterization of liquids tested could be similar, but not identical [43,45,46,47,50,55,60]. Measures of IDDSI flow test may vary depending on the type of thickening agent (starch versus gum) and the liquid used, even though some rheological parameters such as viscosity are similar [43,45,47]. Thus, two liquids of the same level IDDSI may have different viscosities or different flows when measured with another method [43,45,46,60].

#### 3.2.2. Discrimination Ability of the IDDSI Flow Test

The purpose of assessing the ability of the proposed IDDSI syringe to discriminate between levels is to verify whether liquids of different IDDSI levels are distinguishable when consumed in a therapeutic context. Given the complexity of organoleptic food assessment, sensory assessments are regularly used to describe the evolution and perception of food, semi-solid and liquid in the mouth, thus establishing correlations between the texture felt and the texture measured by instrumentation [64]. Thickened lemon-flavored water (starch and gums; with and without barium) was evaluated by panels of trained and untrained healthy subjects. These panels showed that perceived viscosity, slipperiness, ease of swallowing, ease of oral handling of the bolus and impression of adhesion were significantly different between lemon-flavored water samples of different IDDSI levels [48,49]. On the other hand, significant differences were also observed for samples of the same IDDSI level, suggesting that liquids considered similar by IDDSI categorization may have different perceptions in the mouth for some healthy evaluators. Sensory differences could be due to the type of thickening agent used (starch versus gum or type of gum) [48,49,56].

The discrimination capacity of the IDDSI flow test can also be assessed by observing the behavior of liquids during swallowing. Thus, the strength of the tongue and certain parameters observable by videofluoroscopic assessments were measured in healthy adult subjects (age range: 21–58 year.) while swallowing thickened water of different IDDSI Levels [51,52]. Consistent with previous literature [65], the results showed that the force exerted by the tongue during swallowing increased with the consistency of the liquid (*n* = 38) [52]. However, this increase was not proportional in each of the IDDSI levels. In terms of observation of videofluoroscopic parameters, significant differences in pharyngeal response time were demonstrated only between IDDSI grouped Levels 0, 1 and 2 versus IDDSI grouped Levels 3 and 4 (*p* < 0.001) [51]. Similarly, a second study with videofluoroscopic observations in healthy subjects (*n* = 8, age range: 22–30 year.) showed significant differences in pharyngeal transport time only between IDDSI Levels 1 and 4 (*p* < 0.01) [44]. The small sample size is an important limitation in this study.

In 2018, Su et al. published the only study conducted among dysphagic adults (*n* = 26; age range: 53–105 year.) of a rehabilitation unit of Shanghai Huadong Hospital (China) to document the association between the severity of dysphagia and the tolerance of thickened fluids of IDDSI 0–3 levels [42]. Initially, the severity of dysphagia was assessed using a bedside test, the Water Drinking Test. In comparison to videofluoroscopy assessment, the Water Drinking Test showed a sensitivity of 98% and a specificity of 20% to identify the presence of dysphagia in stroke participants (*n* = 45) [66]. Then, liquid tolerance was assessed using the Volume-Viscosity Swallow Test. This test evaluates patients’ ability to swallow bolus of increasing volumes and viscosities. The authors reported a significant correlation between the results of the bedside evaluation and the tolerated IDDSI level, but the study does not assess whether the results of the bedside evaluations differ significantly between all IDDSI levels, as comparisons between the groups were not made (e.g., IDDSI 1 versus IDDSI 2). The authors of this study also selected bolus corresponding to the threshold values of 1 mL, 4 mL, and 8 mL of the IDDSI flow test. However, the IDDSI group dismisses the use of values exactly at the level limits, as these products cannot be clearly classified (FAQ www.iddsi.org). Finally, the small sample size limits the generalization of results.

### 3.3. Reliability

Seven studies were selected because they presented research results related to the reliability of IDDSI methods. Two studies were excluded because they did not calculate any coefficient [67,68].

The repeatability of the IDDSI flow test was assessed in a study where one evaluator measured various thickened liquids in triplicates (water and juice, starches and/or gums) using a rigorous procedure which controlled for temperature and homogeneity of the samples. The reliability coefficient was excellent for this trained evaluator (ICC: 0.99) [60]. In terms of reproducibility, Martinez et al. have shown that rest time post thickening may increase liquid thickness but, no statistical analysis was conducted for this examination [56]. In Barbon et al., the combination of barium powder and thickener led to additional thickening compared to thickened liquids prepared without barium, but the change in temperature over a 3 h time frame had no significant impact in flow measures [53]. A comparison of means was done while an assessment of agreement between methods might have revealed the magnitude of the observed changes [39]. Generalization for all possible thickened beverages is not possible, as tested liquids were primarily thickened water with varying thickening agents or barium content. As for the selection of the syringe, IDDSI recommends to use specifically a “Luer slip tip” 10 mL syringe with a graduation length of 61.5 mL (www.iddsi.org). Finally, studies have shown that the use of syringes inconsistent with these specifications can lead to variations in the results [54,55,57].

The reproducibility of IDDSI framework has recently been evaluated in in vivo context [59]. Participants (*n* = 68, including 30 university students and 12 health professionals) were asked to categorize 21 samples (food and beverages) using IDDSI methods (syringe, spoon, fork, etc.) and information provided on the website. Overall, the classification task was successfully accomplished at 66.7% ± 12.1% (range: 42.9–95.2%). The agreement between the evaluators was moderate (kappa = 0.54), which means that the same product could be categorized into different levels from one evaluator to another. Pairs of samples (*n* = 8), corresponding to each of the IDDSI levels, were evaluated by all participants to determine if they were categorized in the same way. Overall, the pairs of samples at Levels 0-Very Fluid and 7-Regular were the best categorized (kappas = 0.72 and 0.93); samples of Levels 1-Slightly Thick, 2-Mildly Thick, 3-Moderately Thick and 5-Minced and moistened were the least well categorized (kappas = 0.25 to 0.33). Also, the reliability coefficient used does not take into account that errors in classification by evaluators may be more serious than others; for example, the clinical impact might be greater if an evaluator misclassifies a liquid as IDDSI Level 1 instead of IDDSI Level 3 versus the other way around. The use of a weighted kappa would have been more appropriate [39]. Participation to a hands-on workshop did not significantly improve results [58].

## 4. Discussion

The development of nutritional care plans in the presence of dysphagia frequently requires a change in the texture and consistency of food. The standardization of food products offered to individual with dysphagia is often required to ensure a nutritious and safe diet. The framework proposed by IDDSI is derived from a consensus of experts which, while relevant in the development of clinical treatments, represents less robust than evidence-based assessments to develop safe and effective medical guidelines [69].

Despite the importance of metrology for evidence-based practice, this systematic review could not find any studies assessing the validity of IDDSI methods for evaluating the texture of foods for the treatment of dysphagia. In terms of liquid consistency, the results of this systematic review showed little scientific evidence to support the proposed assessment tools. Thickened fluids, in particular, are known to reduce premature leakage to the oropharynx and the risk of penetration and aspiration [20,65,70]. However, their real benefits on the prevention of aspiration pneumonia are still not demonstrated [32,71]. Only one study in this review documented the association between the severity of dysphagia and tolerance to liquids calibrated with the IDDSI flow test for Levels 0–3 in dysphagic participants [42]. The results show a positive and significant correlation between increasing severity of dysphagia and increasing thickness of the fluids. However, contrary to the authors’ conclusion, these results do not demonstrate that the consistency levels proposed by IDDSI are effective tools for treating dysphagia in patients of all clinical settings [42]. This conclusion would have required a larger sample size, including participants from diverse clienteles and to compare two-by-two the IDDSI levels with appropriate statistical tests. In addition, the test used to assess the severity of participants’ dysphagia, the Water Drinking Test, has low specificity which can lead to errors in participants’ classification. Further research, using robust methodological designs and analyses, is needed to examine the validity of the 5-tier IDDSI categorization and established thresholds for liquids.

Comparing related tools can be useful when validating a method. When assessing measurement instruments, the COSMIN assumes that the tools compared have sufficiently documented metrological qualities [39]. Thus, the syringe test was compared to the Bostwick consistometer and the line-spread test methods. However, the validity of these instruments for the measurement of liquids in the treatment of dysphagia has also been very little documented, which limits the interpretation of the results. It is clear that these tools differ in the way they take into account the factors that may influence the measurement of a liquid’s flow, such as adhesion and cohesion, but to date, the role of these factors in the course of swallowing is still under consideration and they can vary greatly depending on the composition of the liquid (pH, nutrients, type of thickening agent, addition of barium, etc.) [20,53,72]. Therefore, the differences between the liquids used in the studies identified in this review, as well as the methods of liquid preparation, make it impossible to generalize the results. Finally, only one study had for main objective to document the reproducibility of IDDSI methods [59]. The results of this review suggest that IDDSI methods would require rigorously designed protocols to confirm their reproducibility.

## 5. Limitations

Although every effort was taken to ensure scientific rigor for this scientific review, there are limitations that should be acknowledged. The articles included in this systematic review were limited to studies found based on the described search strategy. We recognize that there is some potential for bias when including French and English language studies only in a systematic review. Authors of this review did not contact authors of excluded studies that were perceived as presenting methodological flaws or missing data.

## 6. Conclusions

In the frail older population, dysphagia affects food intake and is associated to undernutrition, sarcopenia, sarcopenic dysphagia, and the vicious circle of aspiration pneumonia. Nutritious adapted foods need to be developed and standardized to promote optimal nutritional status. The IDDSI framework and the tools developed by IDDSI were designed to address an important clinical and scientific need which was to have universal terminology of foods and liquids prescribed for the treatment of dysphagia and accessible assessment methods in order to categorize food and beverages. However, IDDSI methods for assessing the texture of foods have not been validated for clinical use. With respect to the IDDSI Flow test, current studies have not provided scientific or clinical evidence that allows for a categorization of liquids responding to significant variations in swallowing function. The study of the metrological qualities of the IDDSI flow test is desirable to establish its scientific credibility. Studies comparing different instruments and sensory and rheological parameters, including a wider variety of foods and beverages (commercial and home) are needed. Recruitment of dysphagic participants of different age groups and clinical profiles is essential to draw conclusions between the measurement of liquids and the physiological effect during swallowing. In the absence of scientifically proven quantitative testing methods, careful screening and assessment of all potential risk factors affecting food intake should be closely monitored by the clinical team and should involve a thorough nutritional assessment and adapted nutritional treatment. In this highly complex area of research, future work must provide objective, quantitative, validated and reliable measurement tools to food and pharmaceutical industries in order to develop efficient food treatment strategies.

## Figures and Tables

**Table 1:** International Dysphagia Diet Standardisation Initiative (IDDSI) levels for food and drinks [38].

Levels	Food	Drinks	IDDSI Flow Test (Volume Remaining in the 10 mL Syringe after 10 s of Flow)
7	Regular/easy to chew		
6	Soft and bite-sized		
5	Minced and moist		
4	Pureed	Extremely thick	10 mL
3	Liquidized	Moderately thick	8–10 mL
2		Mildly thick	4–8 mL
1		Slightly thick	1–4 mL
0		Thin	˂1 mL

**Table 2:** Search Strategy.

Keywords	Databases	Results (*N*_total_ = 135)
**“IDDSI” OR “International Dysphagia Diet Standardisation Initiative” OR “International Dysphagia Diet Standardisation Initiative”**	Medline	42
	Cinahl	29
	Scopus	43
	Proquest Dissertation and Thesis	4
	Proquest	17

**Table 3:** Contextual characteristics of references documenting construct validity and reliability of IDDSI assessment tools.

**(a) Construct Validity Assessment.**						
**Authors**	**IDDSI**	**Country**	**Type of Study/Participants** **(nb and Age)**	**Tested Products**	**T°C**	**Evaluations Completed**
Barbon et al., 2018 [43]	X	Canada	In vitro	Water + ThickenUp^®®^ Water + ThickenUp Clear^®®^	25°	IDDSI Flow Test—IDDSI Levels 0 to 4 Bostwick Consistometer Line-Spread Test
Hadde et al., 2019 [44]	X	China	In vitro In vivo	Water + ThickenUp^®®^ + Barium Water + ThickenUp Clear^®®^ + Barium Water + Hehongchun + Barium	25°	IDDSI Flow Test—IDDSI Levels 1 to 3 Fork Drip Test—IDDSI Level 4 Videofluoroscopy Viscosity Maximal Extensional Viscosity
Hanson et al., 2019 [45]	X	United Kingdom	In vitro Mathematical Model	Water + ThickenUp^®®^ Water + ThickenUp Clear^®®^ Glycerol + Water (Newtonian)	21°	IDDSI Flow Test—IDDSI Levels 1 to 3 Viscosity
Hron et al., 2020 [46]		USA	In vitro Pediatrics	Homemade purees Commercial purees Infants formula thickened with baby cereal	N/A	IDDSI Flow Test—IDDSI Levels 1 to 3 Fork Drip Test—IDDSI Level 4 (milliliter values not provided) Viscosity
Kim et al., 2018 [47]		South Korea	In vitro	Water + Starch and/or gums Xanthan mainly	20°	IDDSI Flow Test—IDDSI Levels 1 to 3 Line-Spread Test
Ong et al., 2018a [48]	X	Canada	In vitro Sensory University students *n* = 29 untrained *n* = 10 trained	Water + Carboxymethyl cellulose gum Water + Xanthan gum Water + Guar gum	25°	IDDSI Flow Test—IDDSI Levels 1 to 3 Viscosity Description and magnitude estimation scaling: Ease of swallowing, slipperiness, perceived viscosity
Ong et al., 2018b [49]	X	Canada	In vitro Sensory University students *n* = 30 untrained *n* = 23 trained	Water + ThickenUp^®®^ Water + ThickenUp^®®^ + Barium Water + ThickenUp Clear^®®^ Water + ThickenUp Clear^®®^ + Barium	25°	IDDSI Flow Test—IDDSI Levels 1 to 3 Viscosity Description of sensory attributes (sweet, salty, lemon, adhesiveness, graininess, slipperiness, perceived viscosity, ease of manipulation and ease of swallowing) Projective Mapping and Ultra-Flash Profile
Redfearn, A. 2019 [50]	X	United Kingdom	In vitro	Water + Thick & Easy™ Clear Water + Thick & Easy™	19.5°	IDDSI Flow Test—IDDSI Levels 1 to 4 Viscosity
Steele et al., 2019a [51]	X	Canada	In vivo *n* = 40 (50% Males) Healthy Mean Age: 34 yr. (21–58 yr.)	Water + ThickenUp Clear^®®^ + Barium (Same samples as Ref. 6 and 10)	≈22°	IDDSI Flow Test—IDDSI Levels 0 to 4 Videofluoroscopy Sip volume Number of swallows per bolus Penetration-Aspiration Scale Timing measures of swallow Bolus location measures
Steele et al., 2019b [52]	X	Canada	In vivo *n* = 40 (50% Males) Healthy Mean Age: 34 yr. (21–58 yr.)	Water + ThickenUp^®®^ Water + ThickenUp^®®^ + Barium Water + ThickenUp Clear^®®^ Water + ThickenUp Clear^®®^ + Barium (Same samples as Ref. 6)	≈22°	IDDSI Flow Test—IDDSI Levels 0 to 3 Lingual manometry Sip volume
Su et al., 2018 [42]	X	China	Clinical *n* = 26 (85% Males) Dysphagia Mean Age: 88 yr. (53–105 yr.)	Water + Ourdiet Swallow	N/A	IDDSI Flow Test—at critical values between IDDSI Levels 0/1, 1/2, 2/3 and 3/4 Viscosity Water Drinking Test Volume-Viscosity Swallow Test
**(b) Reliability Assessment**						
**Authors**	**IDDSI**	**Country**	**Type of Study/Participants** **(nb and Age)**	**Tested Products**	**T°C**	**Evaluations Completed**
Barbon et al., 2019 [53]	X	Canada	In vitro	Water + ThickenUp^®®^ Water + ThickenUp^®®^ + Barium Water + ThickenUp Clear^®®^ Water + ThickenUp Clear^®®^ + Barium	4° ≈22°	IDDSI Flow Test—IDDSI Levels 1 to 3
Dantas et al., 2018 [54]	X	Brazil	In vitro	Water + Xanthan + Maltodextrin Barium + Xanthan + Maltodextrin	≈22°	IDDSI Flow Test—IDDSI Levels 1 to 3 Flow Test with syringe with different technical specifications than IDDSI Flow Test syringe
Garcia et al. 2019 [55]		USA	In vitro	Water, coffee, prune juice and 2% M.F. milk Lyons Ready Care Thick & Easy™ Simply Thick Thick & Easy™ Clear	N/A	IDDSI Flow Test—IDDSI Levels 0 to 4 Flow Test with syringe with different technical specifications than IDDSI Flow Test syringe Line-Spread Test
Martinez et al., 2019 [56]		Spain	Sensory *n* = 23 Healthy Age: 20–70 yr.	Water + ThickenUp^®®^ Water + Visco^®®^ Instant	21°	IDDSI Flow Test—IDDSI Levels 0 to 4 Viscosity Discrimination Tests: Duo-Trio Test Ranking Test
Matsuyama et al., 2020 [57]	X	Japan	In vitro	Glucose syrup + Water (Newtonian) Water + Homemade Starch Water + Toromerin	20°	IDDSI Flow Test—IDDSI Levels 0 to 3 Flow Test with syringe with different technical specifications than IDDSI Flow Test syringe Viscosity
Rule, D., 2019 [58] Rule, D et al. 2020 [59]		USA	In vitro In vivo *n* = 68 Healthy Age: 18–24 yr. (48.5%) 25–34 yr. (33.8%) 35–44 yr. (10.3%) 45–54 yr. (1.5%) 55+ yr. (5.9%)	Food and liquid items of various textures and consistencies Liquid items thickened with SimplyThick^®®^ EasyMix™	23°	IDDSI Levels 0 to 7 IDDSI Flow Test—IDDSI Levels 1 to 4 Spoon Tilt Test Fork Drip Test Fork Pressure Test and Spoon Pressure Test Quiz pre- and post- self-study training Hands-on training Food and liquid classification task
**(c) Construct Validity and Reliability Assessment**						
**Authors**	**IDDSI**	**Country**	**Type of Study/Participants** **(nb and Age)**	**Tested Products**	**T°C**	**Evaluations Completed**
Côté et al., 2019 [60]		Canada	In vitro	Water and juices (apple, orange, cranberry) pre-thickened (Xanthan/Starch)	8°	IDDSI Flow Test—IDDSI Levels 2 and 3 Bostwick Consistometer

IDDSI: ‘X’ One member of the research team listed is associated to the IDDSI Board; ThickenUp^®®^, Nestlé Health Science: Modified cornstarch; ThickenUp Clear^®®^, Nestlé Health Science: Potato maltodextrin, xanthan gum, potassium chloride; Hehongchun: Xanthan gum and potato starch; Ourdiet Swallow (Ourdiet, Guangzhou, China): Xanthan gum and maltodextrine; Visco^®®^ Instant (Smoothfood, Barcelone, Spain): Maltodextrin, xanthan gum, guar gum; Homemade starch: Potato starch (Matsutani Chemical Co, Hyogo, Japan) + Maltodextrin with DE8 (Matsutani Chemical Industry Co, Hyogo, Japan); Toromerin (Nutri Co Ltd., Mie, Japan): Maltodextrin and modified starch; Thick and Easy™: Starch; Thick and Easy™ Clear: Gum; Lyons Ready Care: Starch; Simply Thick: Water, soluble fiber, xanthan gum, glucono delta-lactone, gellan gum, potassium sorbate, calcium chloride, citric acid, sodium citrate, guar gum, pectin; N/A: Not available.
